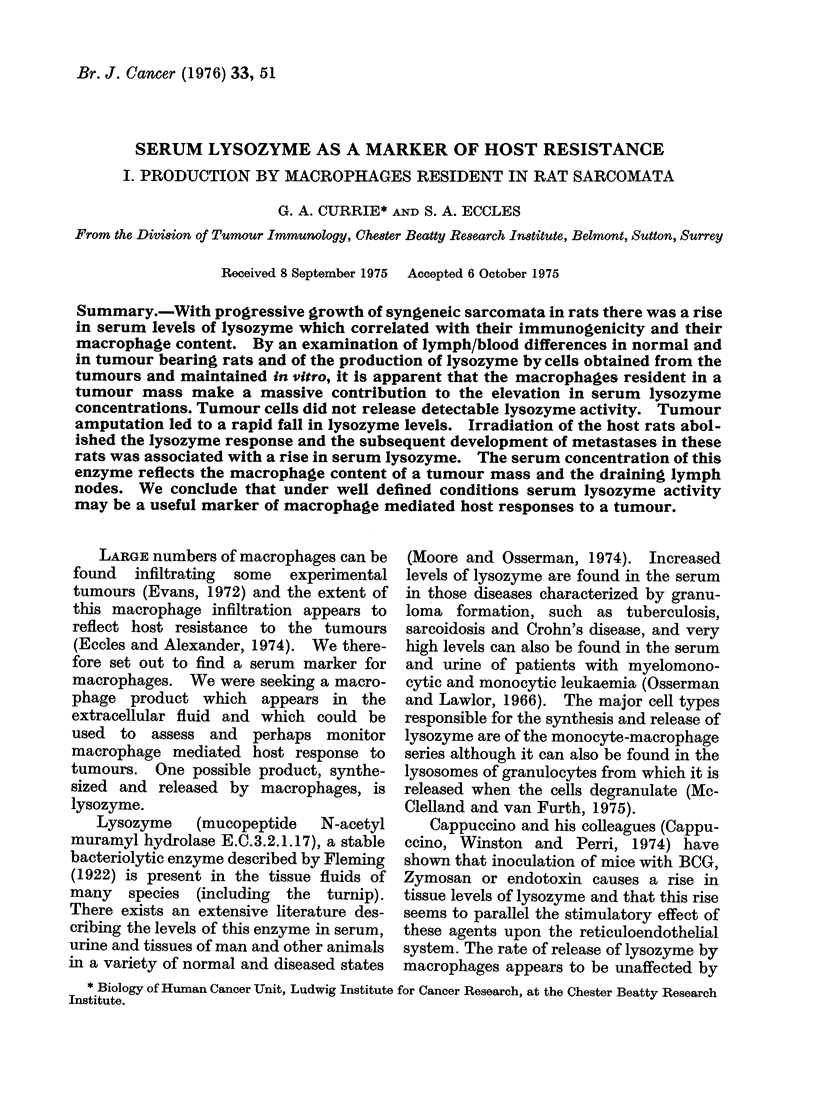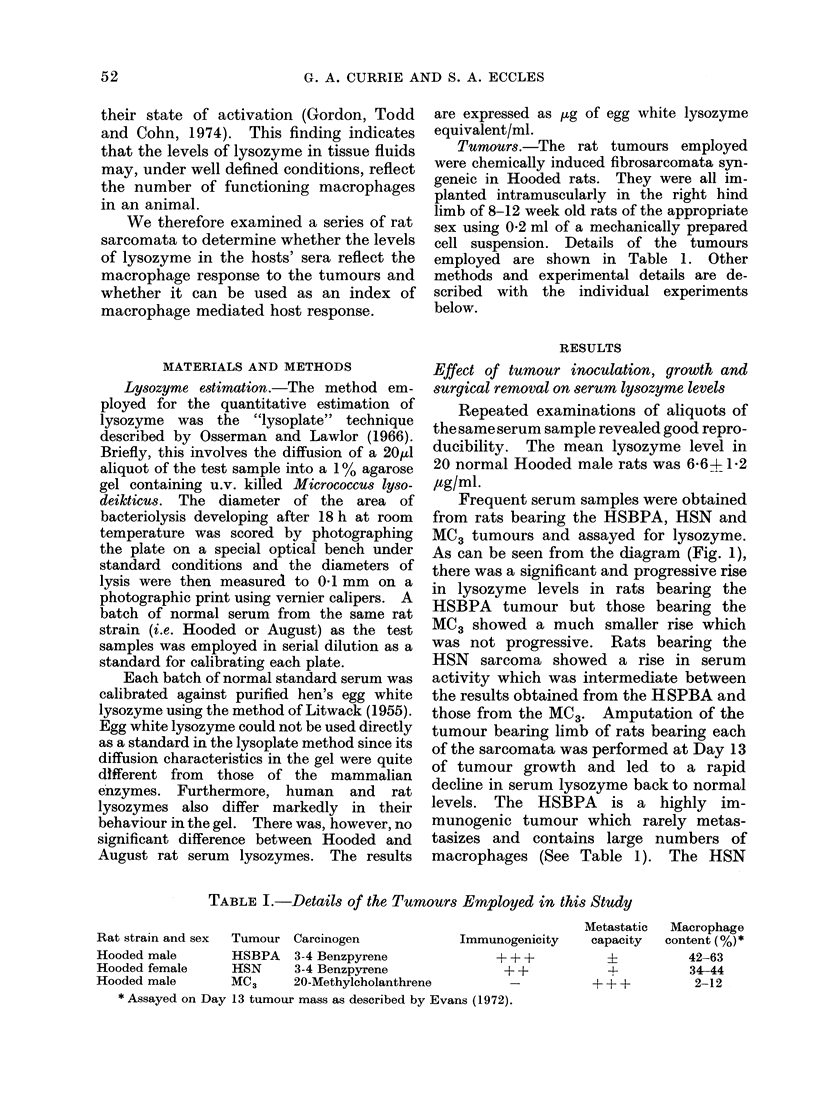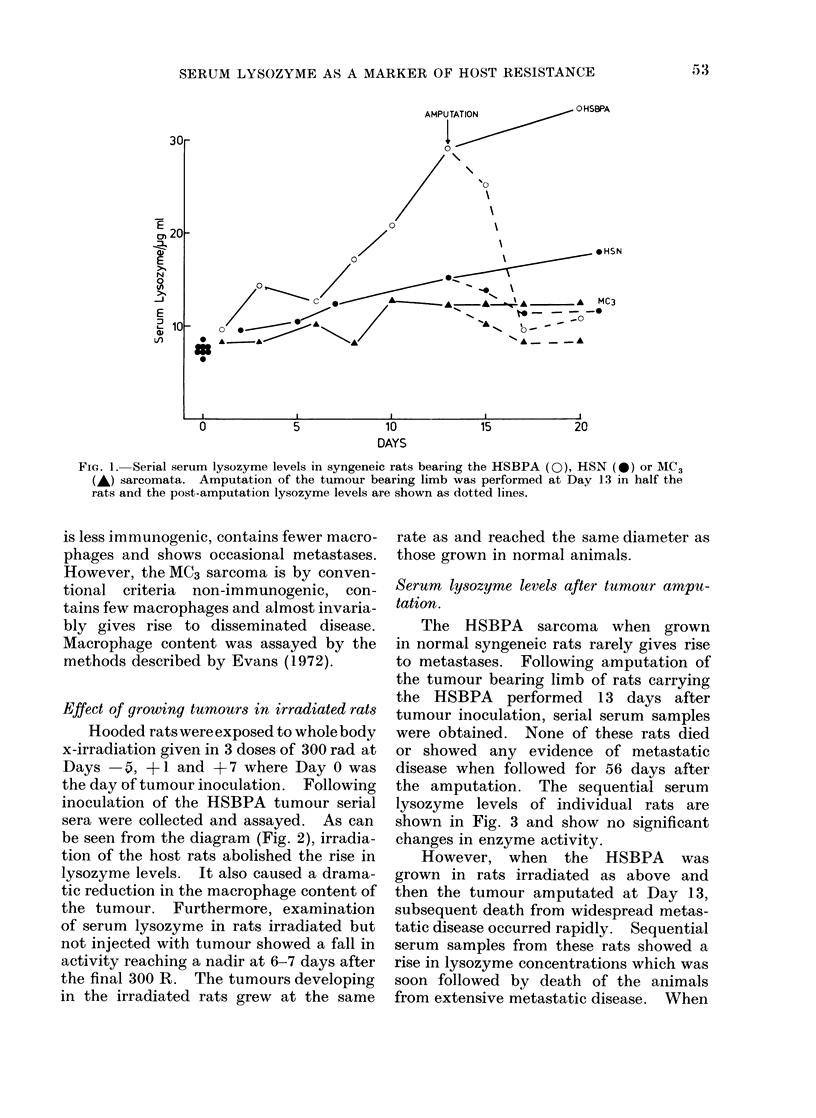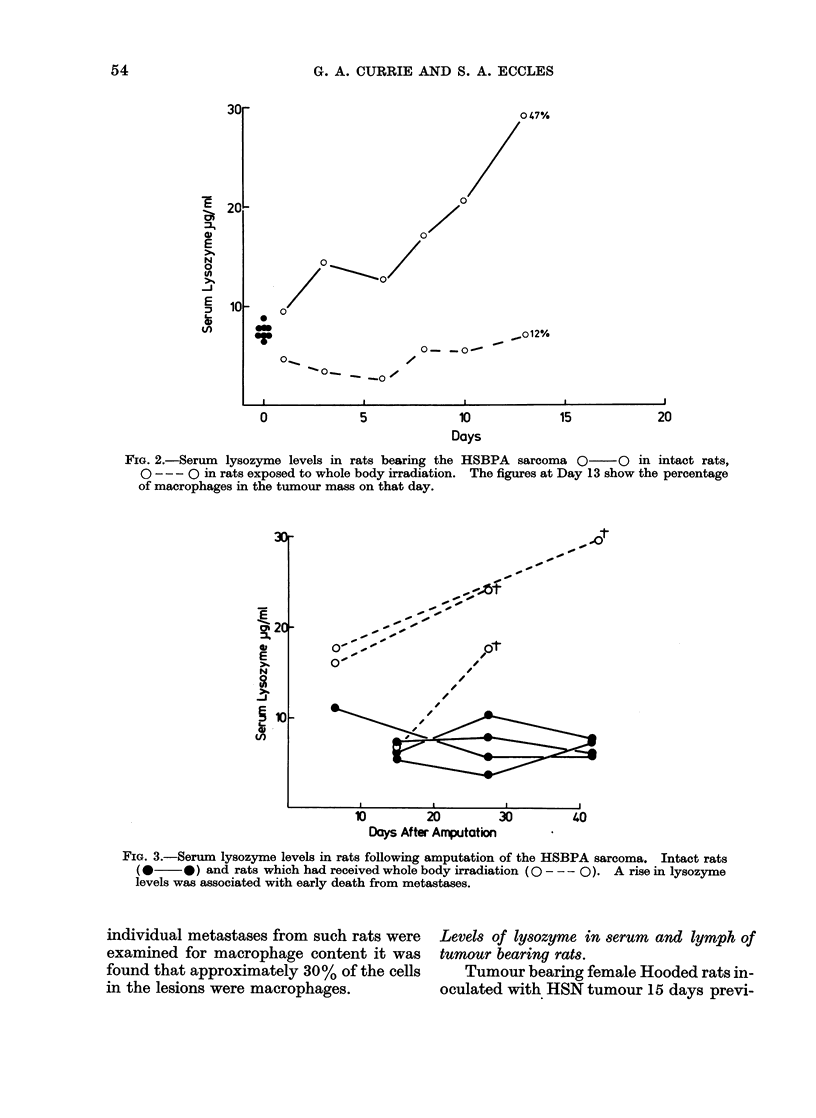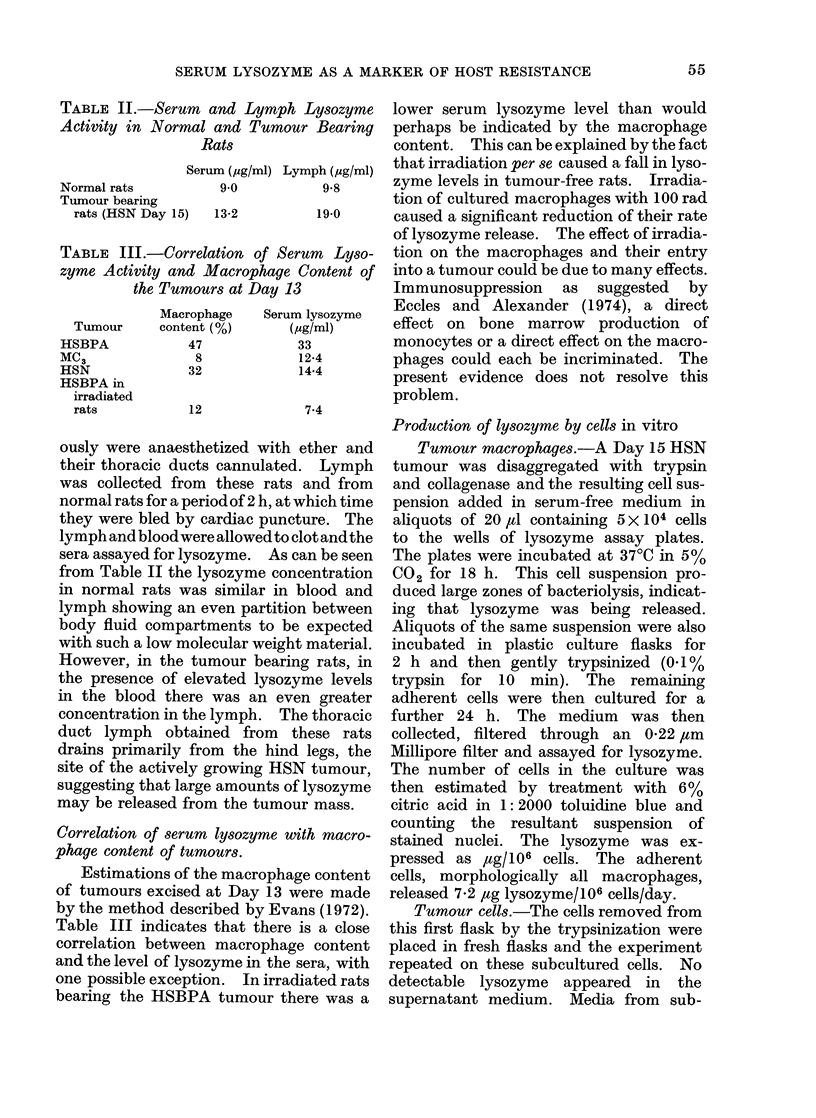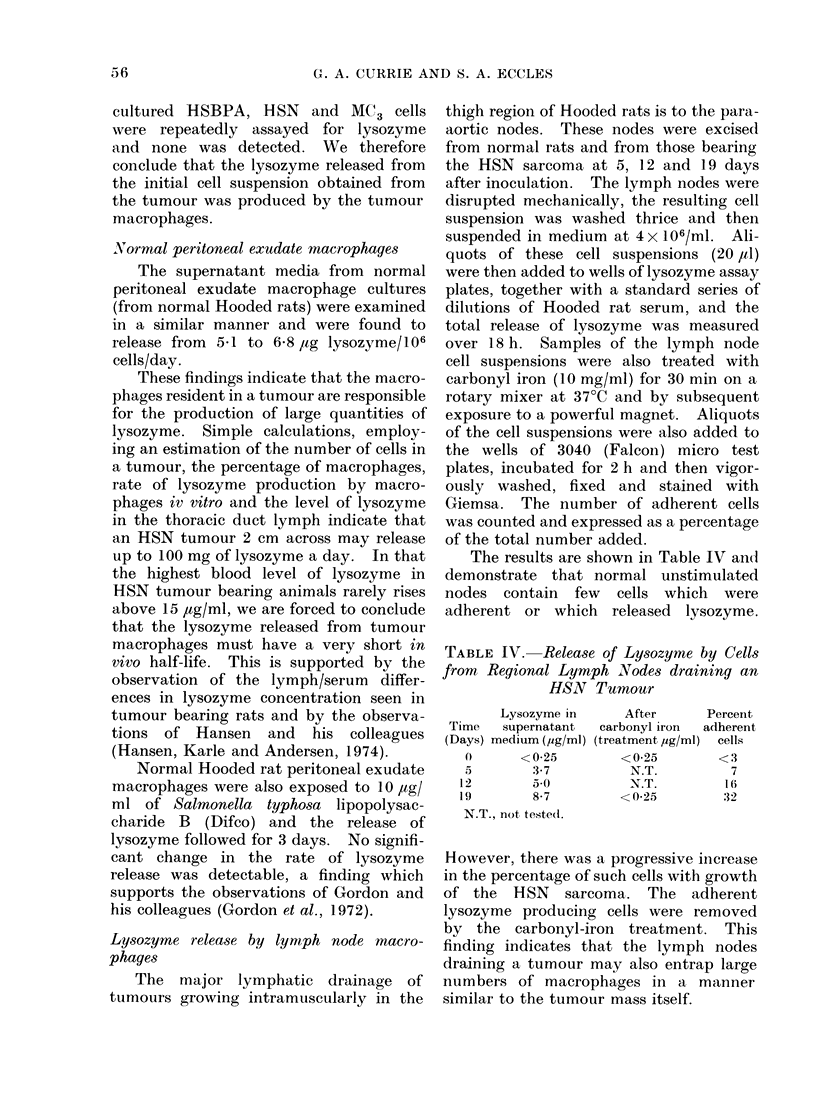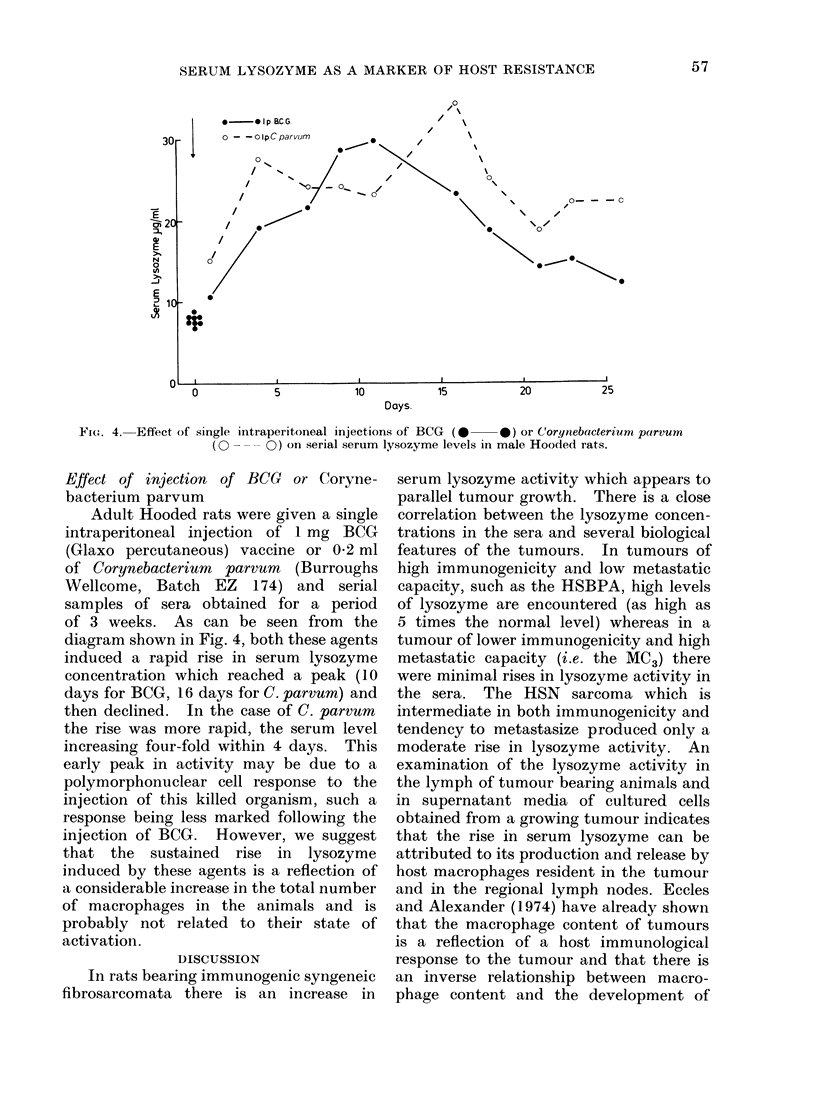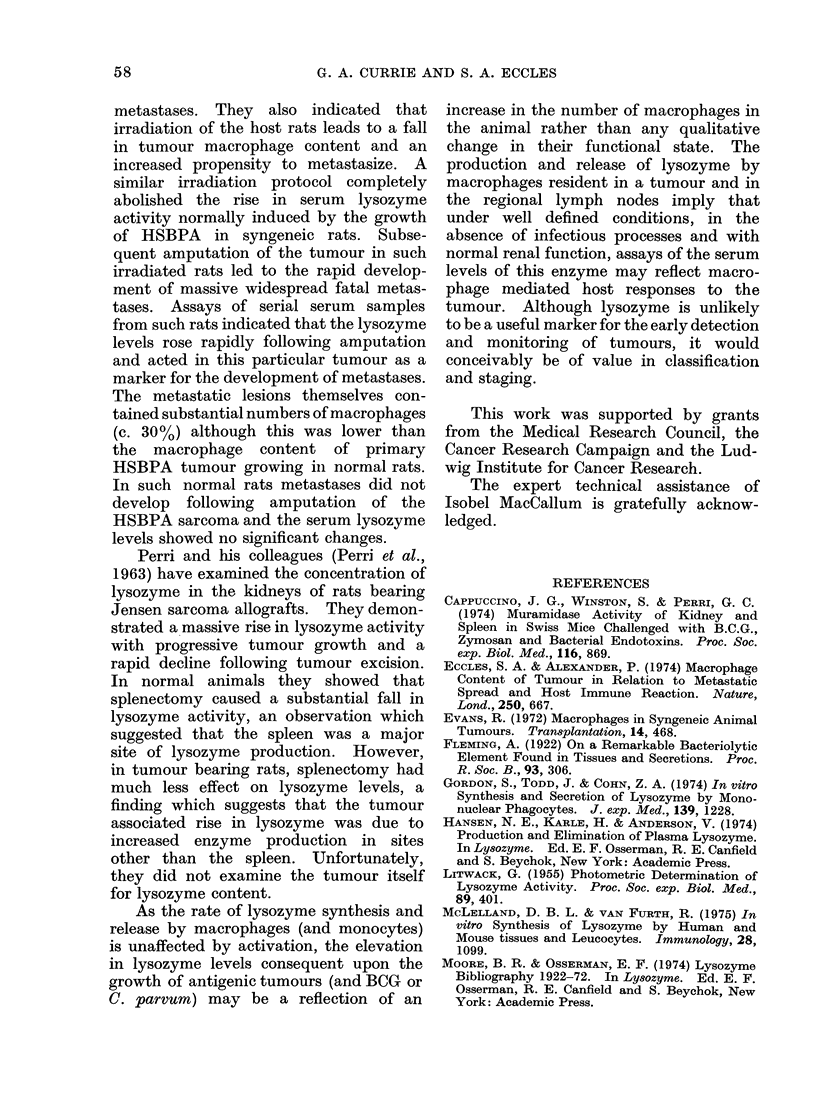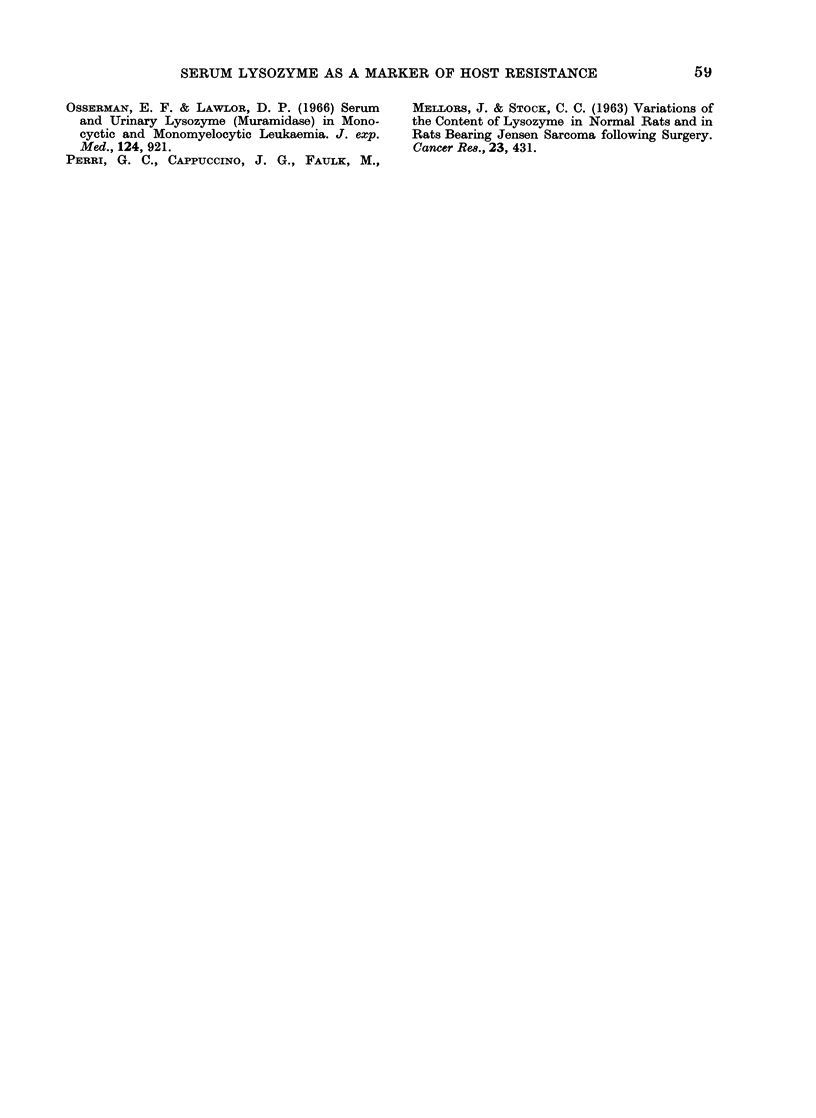# Serum lysozyme as a marker of host resistance. I. Production by macrophages resident in rat sarcomata.

**DOI:** 10.1038/bjc.1976.6

**Published:** 1976-01

**Authors:** G. A. Currie, S. A. Eccles

## Abstract

With progressive growth of syngeneic sarcomata in rats there was a rise in serum levels of lysozyme which correlated with their immunogenicity and their macrophage content. By an examination of lymph/blood differences in normal and in tumour bearing rats and of the production of lysozyme by cells obtained from the tumours and maintained in vitro, it is apparent that the macrophages resident in a tumour mass make a massive contribution to the elevation in serum lysozyme concentrations. Tumour cells did not release detectable lysozyme activity. Tumour amputation led to a rapid fall in lysozyme levels. Irradiation of the host rats abolished the lysozyme response and the subsequent development of metastases in these rats was associated with a rise in serum lysozyme. The serum concentration of this enzyme reflects the macrophage content of a tumour mass and the draining lymph nodes. We conclude that under well defined conditions serum lysozyme activity may be a useful marker of macrophage mediated host responses to a tumour.


Br. J. Cancer (1976) 33, 51

### SERUM LYSOZYME AS A MARKER OF HOST RESISTANCE

### I. PRODUCTION BY MACROPHAGES RESIDENT IN RAT SARCOMATA

G. A. CURRIE* AND S. A. ECCLES

*From the Division of Tumour Immunology, Chester Beatty Research Institute, Belmont, Sutton, Surrey*

Received 8 September 1975 Accepted 6 October 1975

**Summary.—With progressive growth of syngeneic sarcomata in rats there was a rise in serum levels of lysozyme which correlated with their immunogenicity and their macrophage content. By an examination of lymph/blood differences in normal and in tumour bearing rats and of the production of lysozyme by cells obtained from the tumours and maintained *in vitro*, it is apparent that the macrophages resident in a tumour mass make a massive contribution to the elevation in serum lysozyme concentrations. Tumour cells did not release detectable lysozyme activity. Tumour amputation led to a rapid fall in lysozyme levels. Irradiation of the host rats abolished the lysozyme response and the subsequent development of metastases in these rats was associated with a rise in serum lysozyme. The serum concentration of this enzyme reflects the macrophage content of a tumour mass and the draining lymph nodes. We conclude that under well defined conditions serum lysozyme activity may be a useful marker of macrophage mediated host responses to a tumour.**

LARGE numbers of macrophages can be found infiltrating some experimental tumours (Evans, 1972) and the extent of this macrophage infiltration appears to reflect host resistance to the tumours (Eccles and Alexander, 1974). We therefore set out to find a serum marker for macrophages. We were seeking a macrophage product which appears in the extracellular fluid and which could be used to assess and perhaps monitor macrophage mediated host response to tumours. One possible product, synthesized and released by macrophages, is lysozyme.

Lysozyme (mucopeptide N-acetyl muramyl hydrolase E.C.3.2.1.17), a stable bacteriolytic enzyme described by Fleming (1922) is present in the tissue fluids of many species (including the turnip). There exists an extensive literature describing the levels of this enzyme in serum, urine and tissues of man and other animals in a variety of normal and diseased states (Moore and Osserman, 1974). Increased levels of lysozyme are found in the serum in those diseases characterized by granuloma formation, such as tuberculosis, sarcoidosis and Crohn's disease, and very high levels can also be found in the serum and urine of patients with myelomonocytic and monocytic leukaemia (Osserman and Lawlor, 1966). The major cell types responsible for the synthesis and release of lysozyme are of the monocyte-macrophage series although it can also be found in the lysosomes of granulocytes from which it is released when the cells degranulate (McClelland and van Furth, 1975).

Cappuccino and his colleagues (Cappuccino, Winston and Perri, 1974) have shown that inoculation of mice with BCG, Zymosan or endotoxin causes a rise in tissue levels of lysozyme and that this rise seems to parallel the stimulatory effect of these agents upon the reticuloendothelial system. The rate of release of lysozyme by macrophages appears to be unaffected by

* Biology of Human Cancer Unit, Ludwig Institute for Cancer Research, at the Chester Beatty Research Institute.

their state of activation (Gordon, Todd and Cohn, 1974). This finding indicates that the levels of lysozyme in tissue fluids may, under well defined conditions, reflect the number of functioning macrophages in an animal.

We therefore examined a series of rat sarcomata to determine whether the levels of lysozyme in the hosts' sera reflect the macrophage response to the tumours and whether it can be used as an index of macrophage mediated host response.

### MATERIALS AND METHODS

*Lysozyme estimation.*—The method employed for the quantitative estimation of lysozyme was the "lysoplate" technique described by Osserman and Lawlor (1966). Briefly, this involves the diffusion of a 20$\mu$l aliquot of the test sample into a 1% agarose gel containing u.v. killed *Micrococcus lysodeikticus*. The diameter of the area of bacteriolysis developing after 18 h at room temperature was scored by photographing the plate on a special optical bench under standard conditions and the diameters of lysis were then measured to 0·1 mm on a photographic print using vernier calipers. A batch of normal serum from the same rat strain (*i.e.* Hooded or August) as the test samples was employed in serial dilution as a standard for calibrating each plate.

Each batch of normal standard serum was calibrated against purified hen's egg white lysozyme using the method of Litwack (1955). Egg white lysozyme could not be used directly as a standard in the lysoplate method since its diffusion characteristics in the gel were quite different from those of the mammalian enzymes. Furthermore, human and rat lysozymes also differ markedly in their behaviour in the gel. There was, however, no significant difference between Hooded and August rat serum lysozymes. The results are expressed as $\mu$g of egg white lysozyme equivalent/ml.

*Tumours.*—The rat tumours employed were chemically induced fibrosarcomata syngeneic in Hooded rats. They were all implanted intramuscularly in the right hind limb of 8–12 week old rats of the appropriate sex using 0·2 ml of a mechanically prepared cell suspension. Details of the tumours employed are shown in Table 1. Other methods and experimental details are described with the individual experiments below.

### RESULTS

### *Effect of tumour inoculation, growth and surgical removal on serum lysozyme levels*

Repeated examinations of aliquots of the same serum sample revealed good reproducibility. The mean lysozyme level in 20 normal Hooded male rats was 6·6 ± 1·2 $\mu$g/ml.

Frequent serum samples were obtained from rats bearing the HSBPA, HSN and $MC_3$ tumours and assayed for lysozyme. As can be seen from the diagram (Fig. 1), there was a significant and progressive rise in lysozyme levels in rats bearing the HSBPA tumour but those bearing the $MC_3$ showed a much smaller rise which was not progressive. Rats bearing the HSN sarcoma showed a rise in serum activity which was intermediate between the results obtained from the HSPBA and those from the $MC_3$. Amputation of the tumour bearing limb of rats bearing each of the sarcomata was performed at Day 13 of tumour growth and led to a rapid decline in serum lysozyme back to normal levels. The HSBPA is a highly immunogenic tumour which rarely metastasizes and contains large numbers of macrophages (See Table 1). The HSN

TABLE I.—*Details of the Tumours Employed in this Study*

| Rat strain and sex | Tumour | Carcinogen | Immunogenicity | Metastatic capacity | Macrophage content (%)* |
|---|---|---|---|---|---|
| Hooded male | HSBPA | 3-4 Benzpyrene | +++ | ± | 42–63 |
| Hooded female | HSN | 3-4 Benzpyrene | ++ | + | 34–44 |
| Hooded male | $MC_3$ | 20-Methylcholanthrene | — | +++ | 2–12 |

* Assayed on Day 13 tumour mass as described by Evans (1972).

FIG. 1.—Serial serum lysozyme levels in syngeneic rats bearing the HSBPA (○), HSN (●) or $MC_3$ (▲) sarcomata. Amputation of the tumour bearing limb was performed at Day 13 in half the rats and the post-amputation lysozyme levels are shown as dotted lines.

is less immunogenic, contains fewer macrophages and shows occasional metastases. However, the MC3 sarcoma is by conventional criteria non-immunogenic, contains few macrophages and almost invariably gives rise to disseminated disease. Macrophage content was assayed by the methods described by Evans (1972).

*Effect of growing tumours in irradiated rats*

Hooded rats were exposed to whole body x-irradiation given in 3 doses of 300 rad at Days −5, +1 and +7 where Day 0 was the day of tumour inoculation. Following inoculation of the HSBPA tumour serial sera were collected and assayed. As can be seen from the diagram (Fig. 2), irradiation of the host rats abolished the rise in lysozyme levels. It also caused a dramatic reduction in the macrophage content of the tumour. Furthermore, examination of serum lysozyme in rats irradiated but not injected with tumour showed a fall in activity reaching a nadir at 6–7 days after the final 300 R. The tumours developing in the irradiated rats grew at the same rate as and reached the same diameter as those grown in normal animals.

*Serum lysozyme levels after tumour amputation.*

The HSBPA sarcoma when grown in normal syngeneic rats rarely gives rise to metastases. Following amputation of the tumour bearing limb of rats carrying the HSBPA performed 13 days after tumour inoculation, serial serum samples were obtained. None of these rats died or showed any evidence of metastatic disease when followed for 56 days after the amputation. The sequential serum lysozyme levels of individual rats are shown in Fig. 3 and show no significant changes in enzyme activity.

However, when the HSBPA was grown in rats irradiated as above and then the tumour amputated at Day 13, subsequent death from widespread metastatic disease occurred rapidly. Sequential serum samples from these rats showed a rise in lysozyme concentrations which was soon followed by death of the animals from extensive metastatic disease. When

FIG. 2.—Serum lysozyme levels in rats bearing the HSBPA sarcoma ○——○ in intact rats, ○ - - - ○ in rats exposed to whole body irradiation. The figures at Day 13 show the percentage of macrophages in the tumour mass on that day.

FIG. 3.—Serum lysozyme levels in rats following amputation of the HSBPA sarcoma. Intact rats (●——●) and rats which had received whole body irradiation (○ - - - ○). A rise in lysozyme levels was associated with early death from metastases.

individual metastases from such rats were examined for macrophage content it was found that approximately 30% of the cells in the lesions were macrophages.

*Levels of lysozyme in serum and lymph of tumour bearing rats.*

Tumour bearing female Hooded rats inoculated with HSN tumour 15 days previ-

TABLE II.—*Serum and Lymph Lysozyme Activity in Normal and Tumour Bearing Rats*

| | Serum ($\mu$g/ml) | Lymph ($\mu$g/ml) |
|---|---|---|
| Normal rats | 9·0 | 9·8 |
| Tumour bearing rats (HSN Day 15) | 13·2 | 19·0 |

TABLE III.—*Correlation of Serum Lysozyme Activity and Macrophage Content of the Tumours at Day 13*

| Tumour | Macrophage content (%) | Serum lysozyme ($\mu$g/ml) |
|---|---|---|
| HSBPA | 47 | 33 |
| $MC_3$ | 8 | 12·4 |
| HSN | 32 | 14·4 |
| HSBPA in irradiated rats | 12 | 7·4 |

ously were anaesthetized with ether and their thoracic ducts cannulated. Lymph was collected from these rats and from normal rats for a period of 2 h, at which time they were bled by cardiac puncture. The lymph and blood were allowed to clot and the sera assayed for lysozyme. As can be seen from Table II the lysozyme concentration in normal rats was similar in blood and lymph showing an even partition between body fluid compartments to be expected with such a low molecular weight material. However, in the tumour bearing rats, in the presence of elevated lysozyme levels in the blood there was an even greater concentration in the lymph. The thoracic duct lymph obtained from these rats drains primarily from the hind legs, the site of the actively growing HSN tumour, suggesting that large amounts of lysozyme may be released from the tumour mass.

*Correlation of serum lysozyme with macrophage content of tumours.*

Estimations of the macrophage content of tumours excised at Day 13 were made by the method described by Evans (1972). Table III indicates that there is a close correlation between macrophage content and the level of lysozyme in the sera, with one possible exception. In irradiated rats bearing the HSBPA tumour there was a lower serum lysozyme level than would perhaps be indicated by the macrophage content. This can be explained by the fact that irradiation *per se* caused a fall in lysozyme levels in tumour-free rats. Irradiation of cultured macrophages with 100 rad caused a significant reduction of their rate of lysozyme release. The effect of irradiation on the macrophages and their entry into a tumour could be due to many effects. Immunosuppression as suggested by Eccles and Alexander (1974), a direct effect on bone marrow production of monocytes or a direct effect on the macrophages could each be incriminated. The present evidence does not resolve this problem.

*Production of lysozyme by cells* in vitro

*Tumour macrophages.*—A Day 15 HSN tumour was disaggregated with trypsin and collagenase and the resulting cell suspension added in serum-free medium in aliquots of 20 $\mu$l containing $5 \times 10^4$ cells to the wells of lysozyme assay plates. The plates were incubated at 37°C in 5% $CO_2$ for 18 h. This cell suspension produced large zones of bacteriolysis, indicating that lysozyme was being released. Aliquots of the same suspension were also incubated in plastic culture flasks for 2 h and then gently trypsinized (0·1% trypsin for 10 min). The remaining adherent cells were then cultured for a further 24 h. The medium was then collected, filtered through an 0·22 $\mu$m Millipore filter and assayed for lysozyme. The number of cells in the culture was then estimated by treatment with 6% citric acid in 1:2000 toluidine blue and counting the resultant suspension of stained nuclei. The lysozyme was expressed as $\mu g/10^6$ cells. The adherent cells, morphologically all macrophages, released 7·2 $\mu$g lysozyme/$10^6$ cells/day.

*Tumour cells.*—The cells removed from this first flask by the trypsinization were placed in fresh flasks and the experiment repeated on these subcultured cells. No detectable lysozyme appeared in the supernatant medium. Media from subcultured HSBPA, HSN and $MC_3$ cells were repeatedly assayed for lysozyme and none was detected. We therefore conclude that the lysozyme released from the initial cell suspension obtained from the tumour was produced by the tumour macrophages.

*Normal peritoneal exudate macrophages*

The supernatant media from normal peritoneal exudate macrophage cultures (from normal Hooded rats) were examined in a similar manner and were found to release from 5·1 to 6·8 $\mu$g lysozyme/$10^6$ cells/day.

These findings indicate that the macrophages resident in a tumour are responsible for the production of large quantities of lysozyme. Simple calculations, employing an estimation of the number of cells in a tumour, the percentage of macrophages, rate of lysozyme production by macrophages *iv vitro* and the level of lysozyme in the thoracic duct lymph indicate that an HSN tumour 2 cm across may release up to 100 mg of lysozyme a day. In that the highest blood level of lysozyme in HSN tumour bearing animals rarely rises above 15 $\mu$g/ml, we are forced to conclude that the lysozyme released from tumour macrophages must have a very short *in vivo* half-life. This is supported by the observation of the lymph/serum differences in lysozyme concentration seen in tumour bearing rats and by the observations of Hansen and his colleagues (Hansen, Karle and Andersen, 1974).

Normal Hooded rat peritoneal exudate macrophages were also exposed to 10 $\mu$g/ml of *Salmonella typhosa* lipopolysaccharide B (Difco) and the release of lysozyme followed for 3 days. No significant change in the rate of lysozyme release was detectable, a finding which supports the observations of Gordon and his colleagues (Gordon *et al.*, 1972).

*Lysozyme release by lymph node macrophages*

The major lymphatic drainage of tumours growing intramuscularly in the thigh region of Hooded rats is to the para-aortic nodes. These nodes were excised from normal rats and from those bearing the HSN sarcoma at 5, 12 and 19 days after inoculation. The lymph nodes were disrupted mechanically, the resulting cell suspension was washed thrice and then suspended in medium at $4\times10^6$/ml. Aliquots of these cell suspensions (20 $\mu$l) were then added to wells of lysozyme assay plates, together with a standard series of dilutions of Hooded rat serum, and the total release of lysozyme was measured over 18 h. Samples of the lymph node cell suspensions were also treated with carbonyl iron (10 mg/ml) for 30 min on a rotary mixer at 37°C and by subsequent exposure to a powerful magnet. Aliquots of the cell suspensions were also added to the wells of 3040 (Falcon) micro test plates, incubated for 2 h and then vigorously washed, fixed and stained with Giemsa. The number of adherent cells was counted and expressed as a percentage of the total number added.

The results are shown in Table IV and demonstrate that normal unstimulated nodes contain few cells which were adherent or which released lysozyme.

TABLE IV.—*Release of Lysozyme by Cells from Regional Lymph Nodes draining an HSN Tumour*

| Time (Days) | Lysozyme in supernatant medium ($\mu$g/ml) | After carbonyl iron (treatment $\mu$g/ml) | Percent adherent cells |
|---|---|---|---|
| 0 | <0·25 | <0·25 | <3 |
| 5 | 3·7 | N.T. | 7 |
| 12 | 5·0 | N.T. | 16 |
| 19 | 8·7 | <0·25 | 32 |

N.T., not tested.

However, there was a progressive increase in the percentage of such cells with growth of the HSN sarcoma. The adherent lysozyme producing cells were removed by the carbonyl-iron treatment. This finding indicates that the lymph nodes draining a tumour may also entrap large numbers of macrophages in a manner similar to the tumour mass itself.

FIG. 4.—Effect of single intraperitoneal injections of BCG (●——●) or *Corynebacterium parvum* (○ – – – ○) on serial serum lysozyme levels in male Hooded rats.

*Effect of injection of BCG or* Corynebacterium parvum

Adult Hooded rats were given a single intraperitoneal injection of 1 mg BCG (Glaxo percutaneous) vaccine or 0·2 ml of *Corynebacterium parvum* (Burroughs Wellcome, Batch EZ 174) and serial samples of sera obtained for a period of 3 weeks. As can be seen from the diagram shown in Fig. 4, both these agents induced a rapid rise in serum lysozyme concentration which reached a peak (10 days for BCG, 16 days for *C. parvum*) and then declined. In the case of *C. parvum* the rise was more rapid, the serum level increasing four-fold within 4 days. This early peak in activity may be due to a polymorphonuclear cell response to the injection of this killed organism, such a response being less marked following the injection of BCG. However, we suggest that the sustained rise in lysozyme induced by these agents is a reflection of a considerable increase in the total number of macrophages in the animals and is probably not related to their state of activation.

DISCUSSION

In rats bearing immunogenic syngeneic fibrosarcomata there is an increase in serum lysozyme activity which appears to parallel tumour growth. There is a close correlation between the lysozyme concentrations in the sera and several biological features of the tumours. In tumours of high immunogenicity and low metastatic capacity, such as the HSBPA, high levels of lysozyme are encountered (as high as 5 times the normal level) whereas in a tumour of lower immunogenicity and high metastatic capacity (*i.e.* the $MC_3$) there were minimal rises in lysozyme activity in the sera. The HSN sarcoma which is intermediate in both immunogenicity and tendency to metastasize produced only a moderate rise in lysozyme activity. An examination of the lysozyme activity in the lymph of tumour bearing animals and in supernatant media of cultured cells obtained from a growing tumour indicates that the rise in serum lysozyme can be attributed to its production and release by host macrophages resident in the tumour and in the regional lymph nodes. Eccles and Alexander (1974) have already shown that the macrophage content of tumours is a reflection of a host immunological response to the tumour and that there is an inverse relationship between macrophage content and the development of metastases. They also indicated that irradiation of the host rats leads to a fall in tumour macrophage content and an increased propensity to metastasize. A similar irradiation protocol completely abolished the rise in serum lysozyme activity normally induced by the growth of HSBPA in syngeneic rats. Subsequent amputation of the tumour in such irradiated rats led to the rapid development of massive widespread fatal metastases. Assays of serial serum samples from such rats indicated that the lysozyme levels rose rapidly following amputation and acted in this particular tumour as a marker for the development of metastases. The metastatic lesions themselves contained substantial numbers of macrophages (c. 30%) although this was lower than the macrophage content of primary HSBPA tumour growing in normal rats. In such normal rats metastases did not develop following amputation of the HSBPA sarcoma and the serum lysozyme levels showed no significant changes.

Perri and his colleagues (Perri *et al.*, 1963) have examined the concentration of lysozyme in the kidneys of rats bearing Jensen sarcoma allografts. They demonstrated a massive rise in lysozyme activity with progressive tumour growth and a rapid decline following tumour excision. In normal animals they showed that splenectomy caused a substantial fall in lysozyme activity, an observation which suggested that the spleen was a major site of lysozyme production. However, in tumour bearing rats, splenectomy had much less effect on lysozyme levels, a finding which suggests that the tumour associated rise in lysozyme was due to increased enzyme production in sites other than the spleen. Unfortunately, they did not examine the tumour itself for lysozyme content.

As the rate of lysozyme synthesis and release by macrophages (and monocytes) is unaffected by activation, the elevation in lysozyme levels consequent upon the growth of antigenic tumours (and BCG or *C. parvum*) may be a reflection of an increase in the number of macrophages in the animal rather than any qualitative change in their functional state. The production and release of lysozyme by macrophages resident in a tumour and in the regional lymph nodes imply that under well defined conditions, in the absence of infectious processes and with normal renal function, assays of the serum levels of this enzyme may reflect macrophage mediated host responses to the tumour. Although lysozyme is unlikely to be a useful marker for the early detection and monitoring of tumours, it would conceivably be of value in classification and staging.

This work was supported by grants from the Medical Research Council, the Cancer Research Campaign and the Ludwig Institute for Cancer Research.

The expert technical assistance of Isobel MacCallum is gratefully acknowledged.

### REFERENCES

Cappuccino, J. G., Winston, S. & Perri, G. C. (1974) Muramidase Activity of Kidney and Spleen in Swiss Mice Challenged with B.C.G., Zymosan and Bacterial Endotoxins. *Proc. Soc. exp. Biol. Med.*, **116**, 869.

Eccles, S. A. & Alexander, P. (1974) Macrophage Content of Tumour in Relation to Metastatic Spread and Host Immune Reaction. *Nature, Lond.*, **250**, 667.

Evans, R. (1972) Macrophages in Syngeneic Animal Tumours. *Transplantation*, **14**, 468.

Fleming, A. (1922) On a Remarkable Bacteriolytic Element Found in Tissues and Secretions. *Proc. R. Soc. B.*, **93**, 306.

Gordon, S., Todd, J. & Cohn, Z. A. (1974) *In vitro* Synthesis and Secretion of Lysozyme by Mononuclear Phagocytes. *J. exp. Med.*, **139**, 1228.

Hansen, N. E., Karle, H. & Anderson, V. (1974) Production and Elimination of Plasma Lysozyme. In *Lysozyme*. Ed. E. F. Osserman, R. E. Canfield and S. Beychok, New York: Academic Press.

Litwack, G. (1955) Photometric Determination of Lysozyme Activity. *Proc. Soc. exp. Biol. Med.*, **89**, 401.

McLelland, D. B. L. & van Furth, R. (1975) *In vitro* Synthesis of Lysozyme by Human and Mouse tissues and Leucocytes. *Immunology*, **28**, 1099.

Moore, B. R. & Osserman, E. F. (1974) Lysozyme Bibliography 1922–72. In *Lysozyme*. Ed. E. F. Osserman, R. E. Canfield and S. Beychok, New York: Academic Press.

OSSERMAN, E. F. & LAWLOR, D. P. (1966) Serum and Urinary Lysozyme (Muramidase) in Monocytic and Monomyelocytic Leukaemia. *J. exp. Med.*, **124**, 921.
PERRI, G. C., CAPPUCCINO, J. G., FAULK, M., MELLORS, J. & STOCK, C. C. (1963) Variations of the Content of Lysozyme in Normal Rats and in Rats Bearing Jensen Sarcoma following Surgery. *Cancer Res.*, **23**, 431.